# Celiac Artery Compression Syndrome: A Unique Presentation

**DOI:** 10.7759/cureus.16175

**Published:** 2021-07-04

**Authors:** Hasan Ali, Maryam I Kazmi, Jorge A Barajas-Ochoa, Sushil Ahlawat

**Affiliations:** 1 Internal Medicine, Rutgers University New Jersey Medical School, Newark, USA; 2 Gastroenterology and Hepatology, Rutgers University New Jersey Medical School, Newark, USA

**Keywords:** celiac artery compression syndrome, median arcuate ligament syndrome, dunbar syndrome, unintentional weight loss, celiac axis compression syndrome

## Abstract

Celiac artery compression syndrome (CACS), also known as median arcuate ligament syndrome, celiac axis syndrome, and Dunbar Syndrome, is a rare disorder that results from compression of the celiac artery by the median arcuate ligament. The following is a case that depicts an interesting presentation of a patient diagnosed with this rare condition. A 44-year-old male with a history of mutism was brought in by his family for weight loss of 100 lbs with intermittent abdominal pain, weakness and lethargy over a period of five years. His family reported that he had poor nutritional intake, and could only eat a small amount before he seemed to be in pain, and eventually refused to eat. He had no other prior medical history except for mutism, no family history of malignancy, no history of trauma, surgeries, smoking or substance use, and did not take any medications. Physical exam was largely unremarkable. Mesenteric vascular duplex demonstrated severe grade stenosis of the celiac trunk with post-stenotic velocity of 520 cm/sec. Contrast enhanced computed tomography angiography revealed acute angle J-configuration of the takeoff of the celiac axis, with stenosis at its origin and focal post-stenotic dilatation, confirming the diagnosis of CACS.

CACS is an elusive diagnosis that should be considered in patients where other causes of abdominal pain and weight loss have been ruled out. The disease can present with the classic triad of post-prandial abdominal pain, weight loss, and an abdominal bruit. Imaging modalities including mesenteric vascular duplex, computed tomography abdominal angiography, magnetic resonance angiography and celiac artery angiography can help make the diagnosis. Treatment involves surgical decompression via division of the median arcuate ligament, with most patients experiencing significant and long-lasting relief from their symptoms.

## Introduction

Celiac artery compression syndrome (CACS), also known as median arcuate ligament syndrome, celiac axis syndrome, and Dunbar Syndrome, is a rare disorder that results from compression of the celiac artery by the median arcuate ligament [1]. The disorder is diagnosed in only 2 out of 100,000 cases in patients with abdominal pain where other causes have been ruled out. It is more common in females and patients often present at a young age [2,3]. Patients clinically present with the classic triad of post-prandial abdominal pain, weight loss over time, and an abdominal bruit [2,4]. The diagnosis can be made with a combination of imaging modalities including mesenteric vascular duplex, computed tomography angiography, and less commonly celiac artery angiography with respiratory maneuvers [1,2,5-7]. Treatment is achieved primarily through surgical decompression of the celiac trunk via open, laparoscopic or robot-assisted division of the median arcuate ligament, with select patients requiring intravascular procedures to relieve stenosis of the celiac artery [2,3,8]. The following is a case that depicts an interesting presentation of a 44-year-old male diagnosed with this rare condition. 

## Case presentation

A 44-year-old male with past history of mutism was brought in by his family for significant weight loss with associated intermittent abdominal pain, weakness and lethargy over a period of years. Per the family, he used to be overweight four years ago. The patient has been living in Haiti his entire life and recently migrated to the United States one month ago. Per the patient’s family, he has had chronic abdominal pain for five years associated with weight loss of nearly 100 lbs. His family also reported that he had poor nutritional intake; he could only eat a small amount before he seemed to be in pain, and eventually refused to eat. He also exhibited constipation, passing small hard stools once every few days. He had no other prior medical history, no family history of malignancy, no history of trauma or surgeries, no history of smoking or substance use, and did not take any medications. The gastroenterology team was consulted to investigate the etiology of abdominal pain and chronic weight loss. The patient was non-verbal but did follow some commands. When asked to point to the area of pain, he pointed to his epigastrium. He was unable to assist much further in answering questions.

His vital signs were normal. His height was 5’ 8”, weight was 90 lbs, and body mass index (BMI) was 13.7 kg/m^2^. Physical exam revealed a thin cachectic male with a scaphoid abdomen and mild tenderness in the epigastrium. His abdomen was otherwise soft and non-distended with normal bowel sounds, no guarding or rebound tenderness, and Murphy’s sign could not be elicited. No abdominal bruit could be heard on auscultation. His skin was dry with tenting and his mucous membranes were dry. There was no scleral icterus. Cardiopulmonary exam was normal. He was nonverbal, but cooperative and calm, with spontaneous movement observed in all extremities without evidence of focal weakness. There were no signs of skin rash or bruising.

Labs were significant for mild normocytic anemia with hemoglobin 13.1 g/dL. Chemistry panel was normal. Hepatic function panel showed a mildly elevated aspartate aminotransferase and alanine aminotransferase at 60 U/L and 74 U/L, respectively, mildly reduced albumin at 3.4 g/dL and elevated alkaline phosphatase at 199 U/L. Lactic acid, amylase, lipase, erythrocyte sedimentation rate and c-reactive protein were normal. Stool samples were negative for bacteria, viruses, ova and parasites. Abdominal X-ray revealed a normal bowel gas pattern without signs of obstruction. 

**Figure 1:**
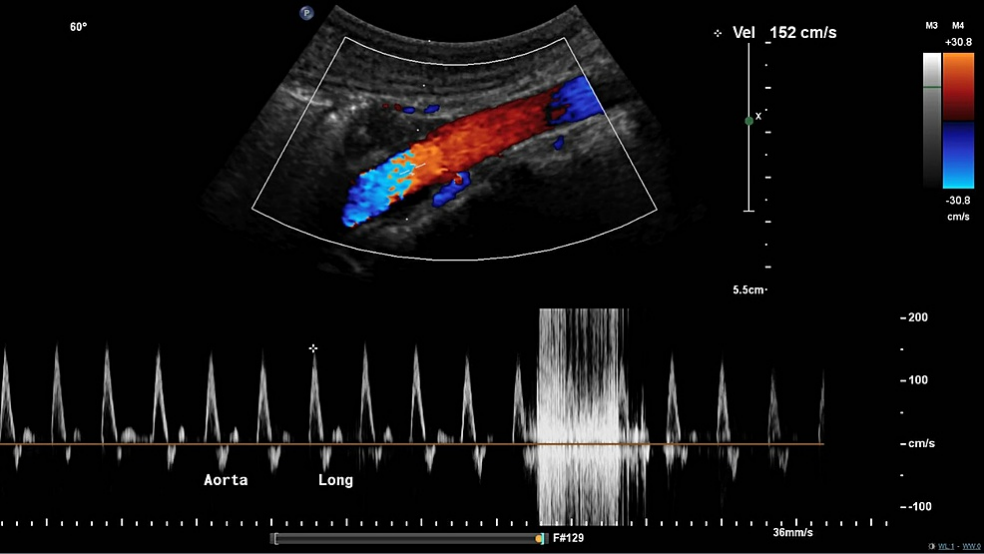
Vascular duplex of abdominal aorta Vascular duplex demonstrated a patent abdominal aorta with a peak systolic velocity of 152 cm/second.

**Figure 2:**
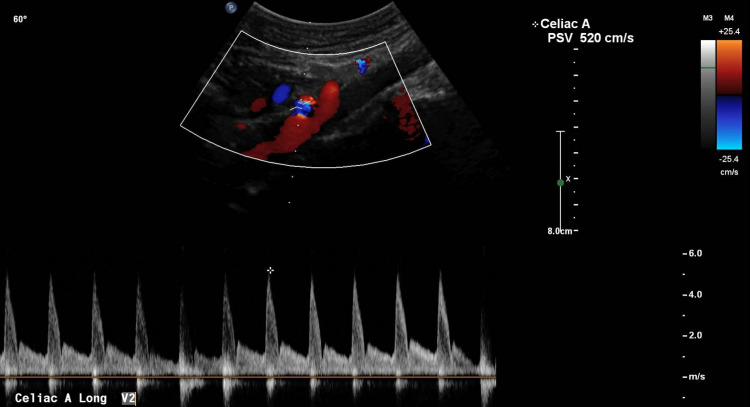
Mesenteric vascular duplex of celiac artery Vascular duplex of the celiac trunk demonstrated a pre-stenosis velocity of 152 cm/second, and a post-stenosis velocity of 520 cm/second with a ratio of post-stenosis velocity to pre-stenosis velocity of >3:1, indicative of severe grade stenosis.

**Figure 3:**
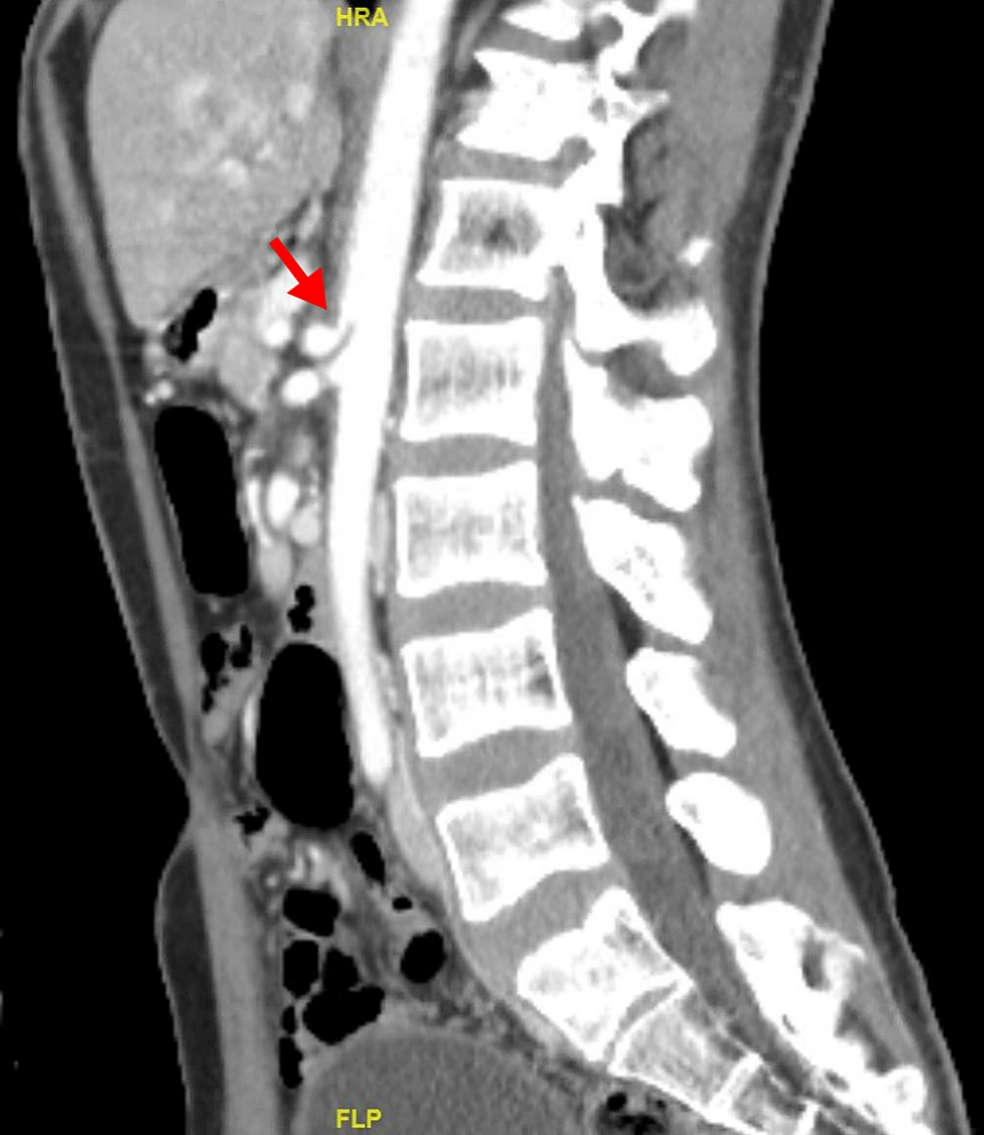
Computed tomography abdominal angiography Computed tomography angiography of the abdomen demonstrated acute angle J-configuration of the takeoff of the celiac axis, with stenosis (red arrow) at its origin and focal post-stenotic dilatation.

Abdominal duplex and computed tomography abdominal angiography were performed (Figures 1-3). 

The mesenteric vascular duplex revealed patency of the abdominal aorta with a peak velocity of 152 cm/second (Figure 1), whereas examination of the celiac trunk demonstrated severe grade stenosis with a pre-stenotic velocity of 152 cm/second and a remarkably high post-stenotic velocity of 520 cm/second (Figure 2). The pre-stenosis velocity to post-stenosis velocity ratio was greater than 3:1. Interestingly, the peak velocity did not vary significantly with respiration, which may have indicated chronic fibrotic changes in the wall of the celiac artery secondary to compression over time. Subsequently, contrast-enhanced computed tomography angiography of the abdomen revealed acute angle J-configuration of the takeoff of the celiac axis, with stenosis (red arrow) at its origin and focal post-stenotic dilatation (Figure 3). Thus, the diagnosis of celiac artery compression syndrome was made. The patient is currently pending evaluation for surgical division of the median arcuate ligament in order to decompress the stenosis at the origin of the celiac artery.

## Discussion

Celiac artery compression syndrome (CACS; also known as median arcuate ligament syndrome, celiac axis syndrome, and Dunbar Syndrome), is a rare disorder that results from compression of the celiac artery by the median arcuate ligament, a fibrous arch that forms at the base of the diaphragm where the right and left diaphragmatic crura join medially to form the anterior portion of the aortic hiatus [1].

This disorder can occur in the setting of anatomic anomalies such as an abnormally cephalad origin of the celiac trunk, or abnormally caudad insertion of the diaphragm [2,3]. Physical extrinsic compression of the celiac trunk by the median arcuate ligament, as well as celiac ganglion plexus dysfunction leading to splanchnic vasoconstriction, is thought to cause intermittent mesenteric ischemia in the area of vascular distribution supplied by branches of the celiac trunk [3]. The external compression is thought to be pronounced during expiration, as cephalad motion of the diaphragm stretches the crura and increases tension at the median arcuate ligament. Conversely, compression may be relieved during inspiration, where caudad movement of the diaphragm increases laxity of the crura. Recurrent compression of the celiac artery over time may lead to fibrotic changes in the arterial wall, resulting in chronic stenosis [2,3].

CACS is a rare disorder, diagnosed in only 2 out of 100,000 cases in patients with abdominal pain where other causes have been ruled out. The disorder has a predilection for females with a ratio of females to males of greater than 3:1 and tends to present in younger patient populations [2,3]. It is estimated that in 10% to 24% of the general population, the median arcuate ligament crosses anterior to the celiac artery, however only a fraction of these patients experience abdominal pain [5,6]. This may be explained in part by the rich collateral blood supply provided by the branches of the superior mesenteric artery [2,5,9].

Although most patients with CACS are asymptomatic, they may clinically present with the classic triad of post-prandial abdominal pain, weight loss over time, and an abdominal bruit on physical examination [2,4]. Symptoms are often associated with nausea, vomiting, dyspepsia and reduced appetite [3]. Post-prandial pain is thought to result from intestinal angina, or increased intestinal oxygen demand that cannot be met due to celiac artery stenosis. However, it is important to note that only 40% of patients with this disorder present with intestinal angina, likely due to sufficient collateral blood supply to the affected region [3,5,9]. Therefore, it is theorized that those without a sufficient collateral blood supply may present with clinical symptoms. Another proposed mechanism is that mechanical irritation of celiac plexus nerve fibers may also contribute to the sensation of abdominal pain [1-3,5].

The diagnosis is challenging to make and is one of exclusion, as a majority of patients are asymptomatic, and because patients’ symptoms are nonspecific and mimic other more common causes of abdominal pain, such as gastritis, peptic ulcer disease, cholecystitis, pancreatitis, and appendicitis. Patients often undergo significant workup for other causes of abdominal pain, and in some cases may undergo unnecessary procedures and surgeries prior to the diagnosis being made [4]. After other causes of abdominal pain are ruled out, the diagnosis can be established by a combination of imaging modalities, as demonstrated in this case. Abdominal vascular duplex with respiratory maneuvers demonstrating greater than or equal to 70 percent stenosis of the celiac artery, a peak expiratory velocity of greater than 350 cm/second, worsening stenosis with expiration, and a deflection angle greater than 50 degrees helps establish the diagnosis [2,5-7]. Contrast-enhanced computerized tomography angiography or magnetic resonance angiography of the abdomen would demonstrate stenosis of the celiac axis secondary to extrinsic compression by the median arcuate ligament, and often post-stenotic dilatation [2,5,6]. The focal narrowing at the origin of the celiac artery often has a J-shaped or hooked appearance, which differentiates it from atherosclerosis or other causes of stenosis [6]. Less commonly utilized is celiac artery angiography with respiratory maneuvers [1,2].

Treatment is achieved primarily through surgical decompression of the celiac trunk via open, laparoscopic or robot-assisted division of the median arcuate ligament, with select patients requiring intravascular procedures to relieve stenosis of the celiac artery [2,3,8]. Patients who undergo surgical decompression often experience marked and lasting symptomatic relief [1,4,8].

## Conclusions

CACS is a rare disorder that results from compression of the celiac artery by the median arcuate ligament. The diagnosis is one of exclusion, as a majority of patients are asymptomatic, and symptoms can mimic those of other more common causes of abdominal pain. CACS should be considered as a differential diagnosis in patients with ambiguous abdominal pain, where other etiologies have been ruled out. Several imaging modalities including mesenteric vascular duplex, computed tomography abdominal angiography, magnetic resonance angiography and celiac artery angiography can help make the diagnosis. Treatment involves surgical decompression via division of the median arcuate ligament, with select patients requiring intravascular procedures to relieve stenosis of the celiac artery. Patients who undergo surgical decompression often have significant and long-lasting relief from their symptoms.
